# Sleep and alertness in shift work disorder: findings of a field study

**DOI:** 10.1007/s00420-018-1386-4

**Published:** 2018-12-03

**Authors:** Päivi Vanttola, Mikko Härmä, Katriina Viitasalo, Christer Hublin, Jussi Virkkala, Mikael Sallinen, Kati Karhula, Sampsa Puttonen

**Affiliations:** 1 Finnish Institute of Occupational Health, Työterveyslaitos, PO Box 40, 00032 Helsinki, Finland; 2 Faculty of Medicine, University of Helsinki, Helsinki, Finland; 3 Finnair Health Services, Finnair, PO Box 15, 01053 Vantaa, Finland; 4 Department of Psychology, University of Jyväskylä, Jyväskylä, Finland

**Keywords:** Insomnia, Sleepiness, Shift work, Circadian rhythm disorders, Sleep diary

## Abstract

**Purpose:**

Although shift work disorder (SWD) affects a major part of the shift working population, little is known about its manifestation in real life. This observational field study aimed to provide a detailed picture of sleep and alertness among shift workers with a questionnaire-based SWD, by comparing them to shift workers without SWD during work shifts and free time.

**Methods:**

SWD was determined by a questionnaire. Questionnaires and 3-week field monitoring, including sleep diaries, actigraphy, the Karolinska Sleepiness Scale (KSS), EEG-based sleep recordings, and Psychomotor Vigilance Tasks (PVT), were used to study 22 SWD cases and 9 non-SWD workers.

**Results:**

The SWD group had a shorter subjective total sleep time and greater sleep debt before morning shifts than the non-SWD group. Unlike the non-SWD group, the SWD group showed little compensatory sleep on days off. The SWD group had lower objective sleep efficiency and longer sleep latency on most days, and reported poorer relaxation at bedtime and sleep quality across all days than the non-SWD group. The SWD group’s average KSS-sleepiness was higher than the non-SWD group’s sleepiness at the beginning and end of morning shifts and at the end of night shifts. The SWD group also had more lapses in PVT at the beginning of night shifts than the non-SWD group.

**Conclusions:**

The results indicate that SWD is related to disturbed sleep and alertness in association with both morning and night shifts, and to less compensatory sleep on days off. SWD seems to particularly associate with the quality of sleep.

**Electronic supplementary material:**

The online version of this article (10.1007/s00420-018-1386-4) contains supplementary material, which is available to authorized users.

## Introduction

In the European Union, 21% of the workforce do some form of shift work (Eurofound 2017) and are potentially exposed to disruption of circadian rhythms. Shift workers are forced to be awake, concentrate, eat, and move against the body’s natural rhythms, which disrupts circadian rhythms and the homeostatic regulation of sleep. The timing of work shifts has an impact on the timing and structure of sleep (Erren et al. 2017). Both night and morning shifts shorten main sleep, reduce the amount of stage R and stage 2 sleep, and are associated with taking naps in the afternoon (Åkerstedt 2003). Sleepiness is common in these shifts (Härmä et al. 2002). Night and evening shifts postpone bedtime, and main sleep after a night shift is typically perceived as insufficient. In comparison, main sleep is cut short before morning shifts and is perceived as unrefreshing (Åkerstedt 2003). All this predisposes employees to various health issues, including insomnia, sleepiness, and shift work disorder (SWD) (Puttonen et al. 2010; Kecklund and Axelsson 2016).

Depending on the studied population, its shift schedules, and the instruments used to define the disorder, as much as 23–63% of shift workers have been reported as having SWD (Waage et al. 2009; Rajaratnam et al. 2011; Flo et al. 2012; Di Milia et al. 2013; Taniyama et al. 2015) based on the International Classification of Sleep Disorders-2 (ICSD-2) criteria. The ICSD-2 defines SWD as insomnia and/or excessive sleepiness that associates with a prolonged shift work schedule which overlaps habitual sleeping time (AASM 2005). The condition should not be better explained by another disorder or medication (AASM 2005). The revised edition, ICSD-3, (1) adds reduced total sleep time (TST) as an accompanying symptom, (2) increases the minimum manifestation time of the shift work schedule-associated symptoms from 1 to 3 months, and (3) increases the duration of sleep diary and actigraphy monitoring (whenever possible), demonstrating a disturbed sleep and wake pattern, from 1 to 2 weeks (AASM 2014).

The ICSD criteria are not particularly specific. As a result, previous studies have defined SWD in various ways. For example, Waage et al. (2009) used three dichotomous questions to define whether the symptoms of SWD associated with a work schedule that overlaps habitual sleeping time. In comparison, Rajaratnam et al. (2011) used a series of questions to define whether night shift-related symptoms of SWD decreased during days off or during daytime. Our questions partially resemble the latter.

Although SWD has been associated with certain shift characteristics, there is limited knowledge regarding how it manifests in different types of shifts in real life. In epidemiological studies, a greater number of night shifts and under 11-h shift intervals have shown to predict SWD among nurses (Flo et al. 2014; Waage et al. 2014). In one field study, shift workers who were dissatisfied with their extremely rapidly backward rotating work schedule were sleepier than their satisfied counterparts during different shifts (Axelsson et al. 2004). Nonetheless, manifestation of SWD in different naturalistic shifts has not been studied in shift work. Research on SWD utilizing shift-specific field data has focused on permanent night work (Gumenyuk et al. 2014, 2015) and consequently lacks data on shift work and morning shifts. The latter is known to be problematic in terms of sleep and alertness (Åkerstedt 2003).

Our aim was to provide a detailed picture of sleep and alertness among shift workers who have questionnaire-based SWD, by comparing them with shift workers without questionnaire-based SWD. We hypothesized that, in association with night and early morning shifts, insomnia symptoms and sleepiness would be more severe among those with SWD than among workers without SWD. In addition, we predicted that the quality of sleep would be poorer among those with SWD.

## Methods

### Study design and setting

**Fig. 1:**
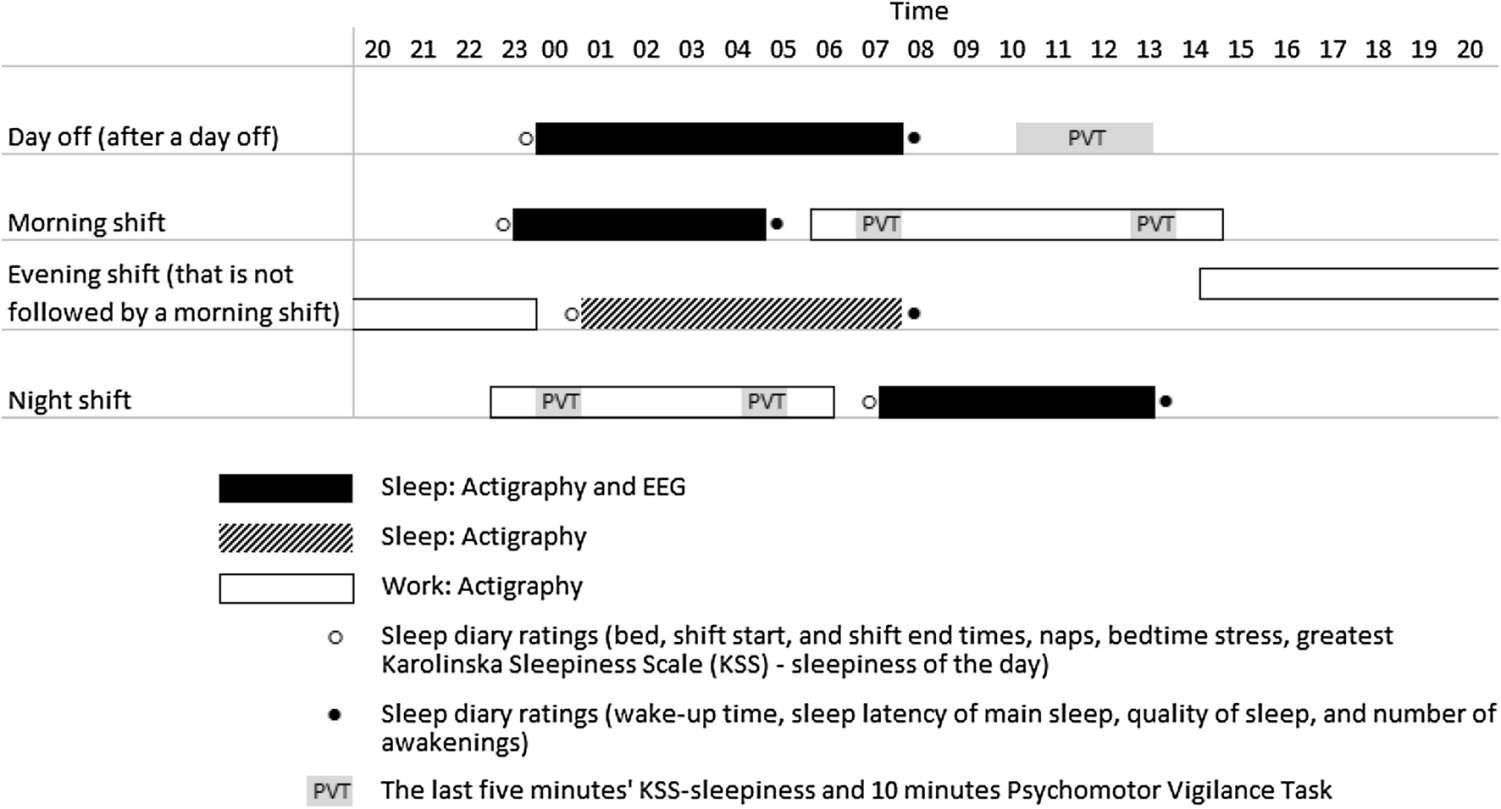
Timing of measurements in relation to shift types and days off

The study included questionnaires and a 3-week observational field study with actigraphy, sleep diaries, Psychomotor Vigilance Tasks (PVT) (Dinges and Powell 1985), ratings of subjective sleepiness (Karolinska Sleepiness Scale, KSS) (Åkerstedt and Gillberg 1990), and EEG-based sleep recordings. Figure 1 presents the timing of the measurements in relation to shift types and days off. The study was carried out between May 2012 and February 2013.

### Participants

The participants were shift workers from the maintenance, customer service, and catering units of a Finnish airline company. The volunteers were recruited on the basis of their responses to a questionnaire of shift-specific questions on insomnia and sleepiness (SS-Q, see ‘Shift work disorder’ section below) in our previous study (*n* = 17) (Viitasalo et al. 2015), in response to an announcement in the company (*n* = 70), or on the advice of occupational health care (*n* = 9).

All volunteers completed a pre-questionnaire on health, medication, working times, and shift-specific questions on insomnia and sleepiness (the SS-Q). They also reported symptoms or a diagnosis of obstructive sleep apnoea (OSA), restless legs syndrome (RLS), and depression. If they had symptoms, occupational health care diagnosed the condition in question. The volunteers were invited to the study if their shift schedule during the past 12 months had regularly included night shifts (at least 3 h of work between 23:00 and 06:00) and/or early morning shifts (starting by 06:00). Those with other shift schedules (*n* = 1), part-time work (*n* = 4), unsuccessful treatment of medically diagnosed OSA or untreated OSA (less than 5 h of continuous positive airway pressure treatment each night) (*n* = 3), RLS and/or depression (*n* = 2), or continual medication affecting sleep (sedatives, antidepressants, or soporific antihistamines) (*n* = 3) were excluded, as were volunteers who changed workplaces (*n* = 1). Volunteers who did not fulfil the pre-set criteria and could not be classified into either the SWD or the non-SWD group during the recruitment process were also excluded (too many holiday-related insomnia symptoms or sleepiness: *n* = 6; not sufficiently often shift-related insomnia symptom(s) and/or sleepiness: *n* = 22; see criteria for the groups in ‘Shift work disorder’ section below). The study included no pregnant or breastfeeding women.

Of the 54 eligible invited volunteers, ten discontinued for health, personal or unknown reasons. In all, 44 completed all the registrations, including a second round of the SS-Q as part of the research questionnaire. As the time interval between answering the SS-Q in the pre-questionnaire and in the research questionnaire was from weeks to months, there was a risk that insomnia symptoms and/or sleepiness may change. Thus, the study groups were formed on the basis of the second answers to the SS-Q. We excluded two participants due to too many holiday-related insomnia symptoms or sleepiness, and a further eleven because they did not have shift-related symptom(s) sufficiently often. Finally, we had 31 participants in our analyses of two groups: 22 in the SWD group (77% working in maintenance, 18% in customer service, and 5% in catering) and nine in the non-SWD group (89% working in maintenance and 11% in customer service). The selection criteria for the groups are presented in the “Shift work disorder”.

### Shift work disorder

Shift work disorder was defined by the SS-Q, which was developed at the Finnish Institute of Occupational Health. The definition is based on the ICSD-2 and is consistent with the updated criteria in the ICSD-3. The SWD symptoms were assessed using six questions in the SS-Q presented in Supplementary table 1 (Online Resource).

Individuals belonged to the SWD group if they often had insomnia symptom(s) and/or sleepiness, which specifically manifested in relation to shifts, but not on holiday. The non-SWD group was composed of those who did not often have insomnia symptoms or sleepiness in relation to shifts or holiday. However, all participants were allowed to have one holiday-related symptom. The symptom that designated a participant as an SWD case could only appear in relation to morning, evening, and/or night shifts, but not in relation to holidays. Supplementary table 1 presents the detailed criteria for the two groups (Online Resource).

According to the ICSD, SWD symptoms should not be better explained by other conditions. To exclude primary conditions with continuing insomnia and/or sleepiness, we did not qualify holiday-related insomnia symptoms and/or sleepiness as indicators of SWD.

### Compliance with ICSD-3

The ICSD-3 specifies that the symptoms of SWD should associate with a recurring work schedule that overlaps the usual time for sleep. The frequency of the symptoms was examined to determine whether the SWD cases’ SWD symptoms, defined by the SS-Q, recurred sufficiently frequently. The questions were part of the online questionnaire and were similar to those of the SS-Q (see Supplementary table 1, Online Resource), but the participants answered on a five-point scale, namely ‘never/less than once a month’, ‘less than once a week’, ‘1–2 times per week’, ‘3–5 times per week’, or ‘daily/almost daily’. All SWD cases reported at least one of the symptoms of insomnia (see Questions 1–4) at least once or twice a week (during the last 3 months) or sleepiness (see Questions 5–6), which occurred on holiday no more than ‘rather rarely’. In addition, as the ICSD-3 specifies TST reduction as a symptom of SWD, we examined the field data to determine whether the average daily TST of each SWD case decreased in comparison to that on days off. Each participant’s daily TST decreased by at least 1 h in relation to morning, evening, and/or night shifts, i.e., both SWD and non-SWD cases. Similarly, as the ICSD specifies that sleep diary and actigraphy monitoring should demonstrate a disturbed sleep and wake pattern, we observed that each SWD case’s timing of sleep changed in relation to the timing of shift type and/or day off.

### Measures

#### Actigraphy and sleep diary

We measured sleep–wake rhythm using a wrist-worn actigraph (Actiwatch AW7, Cambridge Neurotechnology Ltd, Cambs, UK) and a sleep diary. One-minute epochs were analysed using Actiwatch Activity and Sleep Analysis 7 software (Cambridge Neurotechnology Ltd, Cambs, UK). Actigraphy variables included TST, sleep latency, sleep efficiency, and a fragmentation index of sleep.

Participants filled in a sleep diary twice a day, at bedtime and awakening times, and kept a record of bed, wake-up, shift start, and shift end times. The sleep latency of main sleep, bedtime stress scale from 1 (very calm and relaxed) to 9 (extremely stressed and tense), quality of sleep from 1 (good) to 5 (poor), number of awakenings, and the greatest KSS were evaluated daily (Ingre et al. 2004) (Fig. 1).

The TST of main sleep and the preceding naps, based on actigraphy and sleep diaries, were combined as daily TST. Sleep debt was calculated by subtracting daily TST, based on the sleep diary, from sleep need (see ‘Questionnaires’ section below). We averaged the variables based on actigraphy and the sleep diary from the following periods: (1) days off: from bedtime to bedtime prior to and following all days off that were preceded by a day off (the days on which a night shift ended were not considered days off), (2) morning shifts: from bedtime to bedtime prior to and following all morning shifts, (3) evening shifts: from wake-up time to wake-up time prior to and following all evening shifts that were not followed by a morning shift, and (4) night shifts: from wake-up time to wake-up time prior to and following all night shifts.

#### PVT

Participants performed 10-min PVTs that measured vigilant attention on an HP ipaq 514 mobile phone (Hewlett-Packard Company, Palo Alto, CA, USA) (Karhula et al. 2013), and were instructed to practise these three times (Dinges et al. 1997). The PVTs were performed on six pre-selected days: on 2 days off, during two morning shifts starting by 06:00, and during two night shifts. The participants were instructed to complete the PVT in a quiet place with minimal disturbance between 10:00 and 13:00 on days off, and 1–2 h after the start and 1–2 h before the end of the pre-selected shifts (Fig. 1). The PVT variables included mean reaction time and the number of lapses, that is, reaction times of > 500 ms.

#### Subjective sleepiness

Subjective sleepiness was assessed using the nine-point KSS: Participants evaluated their sleepiness (in the last 5 min) prior to each PVT on a mobile phone and their greatest sleepiness each day in their sleep diary (Fig. 1).

#### EEG-based sleep recordings

Sleep EEG was recorded from the forehead using a Zeo Sleep Manager (Zeo, Inc., Newton, MA, USA), a consumer-friendly validated wireless single-channel system (Griessenberger et al. 2013). Participants were asked to sleep one rehearsal night wearing the device before the first recording and to consume no alcohol the day before each recording. Recordings were completed at home at the same pre-selected times as the PVT (Fig. 1): after days off, before morning shifts, and after night shifts. Sleep stages were reported every 30 s. Outcome measures included the TST of main sleep, stage R sleep, light sleep (stages 1–2), and slow-wave sleep (SWS).

#### Questionnaires

Participants completed an online questionnaire including items on demographics, chronotype (1 = absolutely morning type, to 4 = absolutely evening type), flexibility in sleeping habits from the Circadian Type Inventory in the Standard Shift Work Index (scale 8–40, high scores indicate a tendency towards flexibility) (Barton et al. 1995), shift work experience, physical exercise during leisure time (1 = not so much, to 4 = several times a week/competitive type), daily consumption of caffeinated drinks (one dose equals 1 dl of coffee, 2 dl of tea, 5 dl of cola, or 3.3 dl of energy drink), alcohol consumption (1 = never, to 5 = at least 4 times a week), smoking (yes or no), the amount of sleep needed to feel rested the next day (sleep need), number of < 11 h returns to work per month, the SS-Q, and the frequency of SS-Q symptoms.

### Statistical analyses

Work shift characteristics, calculated from roster and sleep diary data, were investigated using the Mann–Whitney *U* test to compare the SWD and non-SWD groups. Questionnaire data were investigated using the independent samples *T* test in cases of normally distributed scaled and continuous variables, the Mann–Whitney *U* test in cases of non-normally distributed continuous variables, and Fisher’s exact test in cases of categorical variables to compare the SWD and non-SWD groups.

Each participant had a unique amount of morning, evening, and night shifts and days off in their field data. To consider this, we conducted linear mixed model analysis (LMM) to compare the groups in cases of normally distributed continuous variables. Due to the difference between the ages of the groups, we used Age, in addition to Group as the main effect in the LMMs. Similarly, we performed LMMs using Chronotype, in addition to Group as the main effect. However, the results for the latter are not shown, because they were similar to the results of the LMM adjusted for age. Prior to model fitting, variables were transformed to meet the model assumptions, where necessary (√*x*: EEG-based TST of main sleep on days off, stage R sleep after night shifts, SWS on days off, SWS after night shifts, and fragmentation index of sleep after evening shifts; √(*x*_max_ + 1 − *x*): light sleep before morning shifts, sleep efficiency on days off, sleep efficiency after evening shifts, and sleep efficiency after night shifts; log_10_x: SWS before morning shifts; log_10_(*x*_max_ + 1 − *x*): sleep efficiency before morning shifts; and *x*^0.7^: fragmentation index of sleep before morning shifts). We applied the Mann–Whitney *U* test when transformations of repeated scaled and continuous variables were unsuccessful. We calculated the means, standard deviations (SD), medians, interquartile ranges (IQR), and the *U* test values of repeated measures from each participant’s averaged values. We used IBM SPSS Statistics 20.0 for the analyses.

## Results

### Sample characteristics

Table 1 presents the sample characteristics. The SWD and non-SWD groups were comparable in terms of sex, chronotype, shift work experience, frequency of physical exercise, alcohol consumption, smoking, and having successfully treated OSA. Individuals with SWD were younger [mean difference = − 6.6 years, 95% CI = (− 13.1, − 0.2)], had less flexibility in their sleeping habits [mean difference = − 6.2, 95% CI = (− 10.6, − 1.9)], and consumed less caffeinated drinks (*U* = 50.00, *p* = 0.032) than the non-SWD group. Supplementary table 2 presents the participants’ work shift characteristics, which did not significantly differ between the groups (*p*s ≥ 0.185, Mann–Whitney *U* tests, Online Resource). In the research questionnaire, the SWD and non-SWD groups reported similar amounts of < 11 h returns to work each month [median (IQR): 0.0 (0.5) and 0.0 (0.0), respectively].

**Table 1:** Sample characteristics

	SWD group (*n* = 22)	Non-SWD group (*n* = 9)	*p*
Age (years), mean (SD)	41 (8)	48 (7)	0.04^a^
Sex (men), % (*n*)	77 (17)	78 (7)	1.00^b^
Chronotype (morning type), % (*n*)	36 (8)	56 (5)	0.43^b^
Flexibility in sleeping habits, mean (SD)	26 (6)	33 (4)	< 0.01^a^
Shift work experience (years), mean (SD)	17 (8)	23 (13)	0.20^a^
Physical exercise (≥ 3 h × week), % (*n*)	50 (11)	22 (2)	0.29^b^
Daily doses of caffeinated drinks, median (IQR)	4 (5)	5 (2)	0.03^c^
Alcohol consumption (≥ 2 × month), % (*n*)	50 (11)	67 (6)	0.46^b^
Smoker, % (*n*)	14 (3)	11 (1)	1.00^b^
Obstructive sleep apnoea, % (*n*)	5 (1)	11 (1)	0.50^b^

^a^Independent samples *t* test

^b^Fisher’s exact test

^c^Mann–Whitney *U* test

### Quantity of sleep

The SWD group reported a longer sleep need in the questionnaire than the non-SWD group [MD (IQR): 8:00 (2:00) and 7:00 (2:00), respectively, *U* = 50.0, *p* = 0.026]. Table 2 presents the results of the LMM analysis of sleep quantity that was evaluated using both subjective (sleep diary) and objective (actigraphy and EEG based) measures. Compared to the non-SWD group, those with SWD reported shorter subjective daily TST in relation to morning shifts (Group: *F*_22.258_ = 4.94, *p* = 0.037). Those with SWD had greater sleep debt on morning shift days and slept less compensatory sleep on days off than those without SWD, as indicated by the LMM analysis of sleep debt before morning shifts (Group: *F*_26.834_ = 11.13, *p* < 0.01) and on days off (Group: *F*_25.567_ = 7.44, *p* = 0.011). The objective TST of the groups did not differ.

**Table 2:** Quantity of sleep among shift workers with and without SWD

	SWD group		Non-SWD group		Group *p* ^a^
	Mean (SD)	*n*	Mean (SD)	*n*	
Sleep debt^b^ (h:mm)					
Days off	− 0:05 (1:08)	22	− 1:32 (1:36)	8	0.01
Before morning shifts	1:58 (0:50)	22	0:23 (1:49)	8	< 0.01
After evening shifts	0:54 (1:19)	22	−0:09 (1:20)	9	0.07
After night shifts	1:36 (1:22)	18	0:51 (1:08)	8	0.14
Daily TST (h:mm)—sleep diary					
Days off	7:58 (1:02)	22	8:17 (0:48)	8	0.49
Before morning shifts	5:55 (0:46)	22	6:30 (0:37)	8	0.04
After evening shifts	6:59 (1:10)	22	7:03 (1:08)	9	0.90
After night shifts	6:16 (1:15)	18	5:54 (1:20)	8	0.49
Daily TST (h:mm)—actigraphy					
Days off	7:19 (0:54)	22	7:58 (0:55)	9	0.17
Before morning shifts	5:37 (0:49)	22	6:08 (0:43)	8	0.20
After evening shifts	6:17 (0:59)	22	6:45 (0:59)	9	0.31
After night shifts	5:48 (1:07)	18	5:46 (0:59)	8	0.98
TST of main sleep (h:mm)—EEG-based recordings					
Days off	7:05 (1:23)	22	8:02 (0:46)	9	0.10
Before morning shifts	5:34 (0:58)	22	5:53 (0:41)	8	0.67
After night shifts	5:02 (1:29)	17	4:55 (1:36)	8	0.64

*TST* total sleep time, *EEG* electroencephalography

^a^Linear mixed model for repeated measurements with Group and Age as main effects

^b^Sleep debt calculated by subtracting daily TST, based on sleep diary, from sleep need. Negative values interpreted as compensatory sleep

The amount of light sleep was shorter on days off among those with SWD than among those without SWD in the LMM analysis of the EEG-based recordings of sleep stages (3:51 ± 1:05 and 4:54 ± 1:07, respectively, Group: *F*_28.844_ = 6.02, *p* = 0.020). Otherwise, the SWD and non-SWD groups did not differ in terms of sleep stages in any shift type or on days off (Group: light sleep: *p* ≥ 0.14; stage R sleep: *p* ≥ 0.27; SWS: *p* ≥ 0.45).

### Quality of sleep

The results of the LMM analysis of actigraphy-based sleep quality are as follows. The SWD group had significantly lower sleep efficiency than the non-SWD group, or showed a trend towards significance (on days off: 89 ± 5 and 94 ± 2%, Group: *F*_29.041_ = 11.91, *p* < 0.01; before morning shifts: 87 ± 6 and 93 ± 4%, Group: *F*_27.304_ = 11.84, *p* < 0.01; after evening shifts: 89 ± 4 and 94 ± 3%, Group: *F*_25.924_ = 13.66, *p* < 0.01; and after night shifts: 90 ± 4 and 93 ± 3%, Group: *F*_22.373_ = 3.73, *p* = 0.066, respectively). In addition, supplementary table 3 presents fragmentation index of sleep among shift workers with and without SWD (Online Resource).

Table 3 presents the results of the objective and subjective sleep quality variables. Compared to the non-SWD group, those with SWD reported significantly poorer quality of sleep across all days (on days off: *U* = 17.5, *p* < 0.001; before morning shifts: *U* = 13.5, *p* < 0.001; after evening shifts: *U* = 21.5, *p* < 0.001; and after night shifts: *U* = 18.0, *p* < 0.01) and longer subjective sleep latency on days off (*U* = 49.0, *p* = 0.029), before morning shifts (*U* = 31.0, *p* < 0.01), and after night shifts (*U* = 21.5, *p* < 0.01). They also showed a trend towards significantly longer subjective sleep latency after evening shifts (*U* = 59.5, *p* = 0.084). Similarly, based on the actigraphy recordings, those with SWD had significantly longer objective sleep latency before morning shifts (*U* = 42.0, *p* = 0.031), after evening shifts (*U* = 48.0, *p* = 0.026), and after night shifts (*U* = 35.0, *p* = 0.040), and showed a trend towards significantly longer objective sleep latency on days off (*U* = 56.5, *p* = 0.064).

**Table 3:** Quality of objective and subjective sleep among shift workers with and without SWD

	SWD group		Non-SWD group		*p* ^a^
	Median (IQR)	*n*	Median (IQR)	*n*	
Number of awakenings—sleep diary					
Days off	2.3 (2.0)	22	1.5 (1.8)	8	0.27
Before morning shifts	2.0 (1.6)	22	1.7 (1.2)	8	0.20
After evening shifts	1.3 (1.9)	22	0.7 (1.3)	9	0.34
After night shifts	1.4 (1.1)	18	0.9 (1.1)	8	0.19
Quality of sleep^b^—sleep diary					
Days off	2.7 (0.9)	22	1.3 (0.4)	8	< 0.001
Before morning shifts	3.0 (0.9)	22	1.6 (0.8)	8	< 0.001
After evening shifts	2.3 (1.0)	22	1.0 (0.4)	9	< 0.001
After night shifts	2.5 (1.4)	18	1.5 (0.9)	8	< 0.01
Sleep latency (min)—sleep diary					
Days off	16 (8)	22	8 (11)	8	0.03
Before morning shifts	20 (34)	22	9 (10)	8	< 0.01
After evening shifts	12 (8)	22	8 (11)	9	0.08
After night shifts	11 (8)	18	5 (4)	8	< 0.01
Sleep latency (min)—actigraphy					
Days off	8 (7)	22	4 (5)	9	0.06
Before morning shifts	6 (8)	22	3 (6)	8	0.03
After evening shifts	8 (12)	22	3 (6)	9	0.03
After night shifts	3 (4)	18	2 (3)	8	0.04

^a^Mann–Whitney *U* test

^b^From 1 (good) to 5 (poor)

In addition to poorer sleep quality, the shift workers with SWD reported higher points on the bedtime stress scale than the non-SWD group [MD (IQR): on days off: 2 (1) and 1 (1), *U* = 26.0 *p* < 0.01; before morning shifts 3 (3) and 1 (1), *U* = 24.0, *p* < 0.01; after evening shifts: 3 (2) and 1 (2); *U* = 39.5 *p* < 0.01; and after night shifts: 3 (2) and 2 (3), *U* = 35.5, *p* = 0.042, respectively].

### Subjective sleepiness and objective alertness

**Fig. 2:**
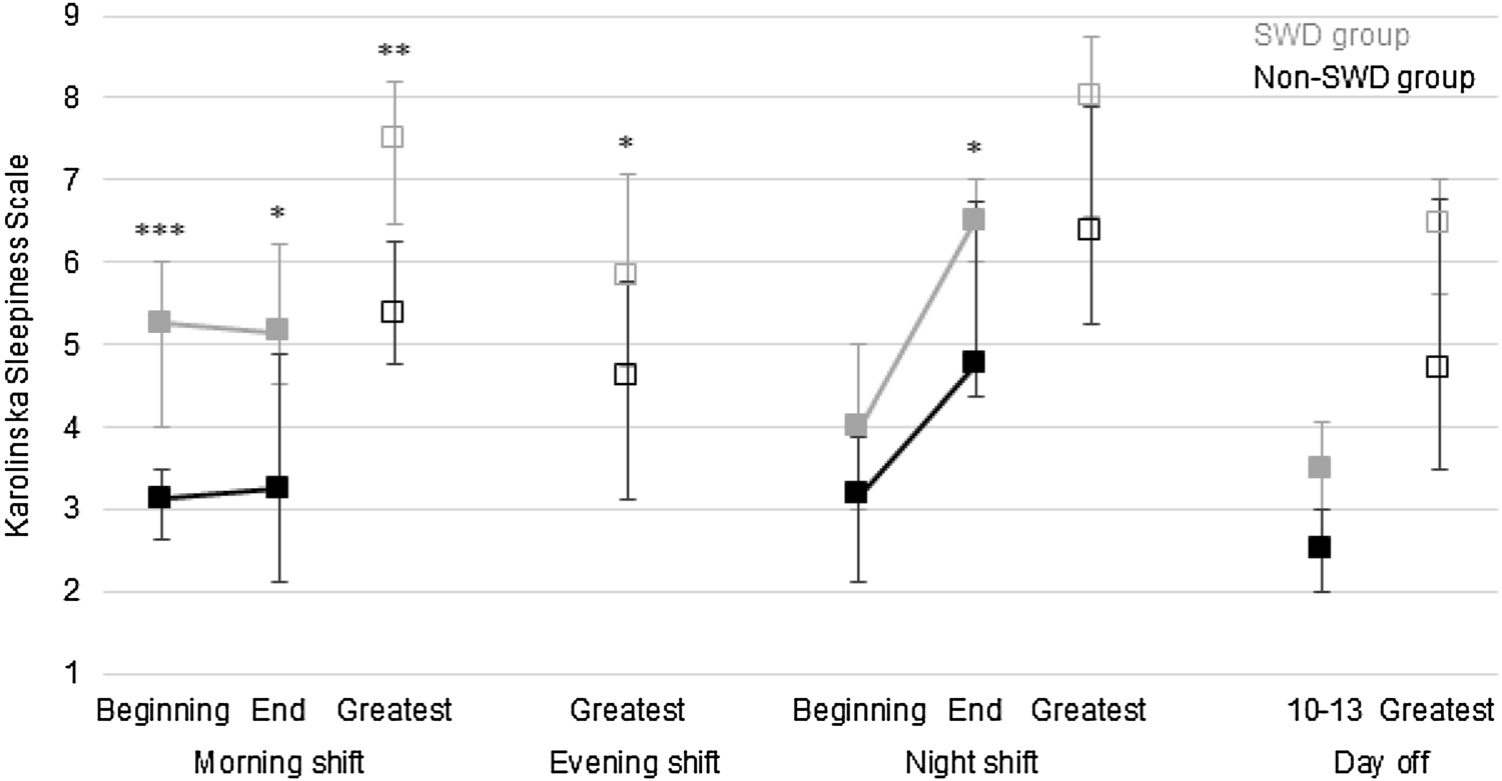
Group medians (with IQR) of subjective sleepiness. Filled square: last 5-min KSS at the beginning or the end of shifts, or between 10:00 and 13:00 on days off. Open square: the greatest daily KSS according to sleep diary. Mann–Whitney *U* test: *p < 0.05; **p < 0.01; ***p < 0.001

Figure 2 presents the group means of subjective sleepiness that were evaluated using two types of measures: the last 5-min KSS and the greatest daily KSS. The Mann–Whitney *U* tests indicated a trend towards greater sleepiness among the shift workers with SWD on days off (KSS between 10:00 and 13:00: *U* = 55.0, *p* = 0.053; greatest daily KSS: *U* = 47.0, *p* = 0.093) than among those without SWD. Similarly, those with SWD had greater sleepiness, or showed a trend towards it, in connection with morning shifts (KSS at the beginning and the end of the shifts: *U* = 17.0, *p* < 0.001 and *U* = 36.0, *p* = 0.014, respectively; greatest daily KSS: *U* = 30.5, *p* < 0.01), evening shifts (greatest daily KSS: *U* = 52.5, *p* = 0.043), and night shifts (KSS at the beginning and end of shifts: *U* = 39.0, *p* = 0.065 and *U* = 30.0, *p* = 0.018, respectively; greatest daily KSS: *U* = 41.5, *p* = 0.088) than the non-SWD group.

Table 4 presents the group means of lapses in PVT. The shift workers with SWD had significantly more lapses in PVT at the beginning of night shifts than those without SWD (*U* = 39.0, *p* = 0.044). The mean reaction times of the groups did not significantly differ in any shift type or on days off (*p*s ≥ 0.18, data not shown).

**Table 4:** Lapses in PVT among shift workers with and without SWD

	SWD group		Non-SWD group		*p* ^a^
	Median (IQR)	*n*	Median (IQR)	*n*	
Days off (10:00–13:00)	0.0 (1.0)	21	0.5 (1.3)	9	0.77
Beginning of morning shifts	0.7 (1.3)	21	0.5 (0.9)	8	0.27
End of morning shifts	0.5 (1.3)	21	0.1 (2.0)	8	0.19
Beginning of night shifts	0.5 (0.6)	18	0.0 (0.0)	8	0.04
End of night shifts	1.0 (4.4)	18	0.8 (3.1)	8	0.61

^a^Mann–Whitney *U* test

## Discussion

We studied the characteristics of sleep and alertness in SWD in various shifts and during free time utilizing information on sleep quantity and quality, sleepiness, and alertness. To our knowledge, this is the first study to investigate SWD in naturalistic morning, evening, and night shifts using both actigraphy and sleep diaries, or to verify disturbed sleep and wake patterns typical to the disorder, as required by the ICSD-3. To ensure that SWD symptoms were related to the shift work schedule, we only qualified as SWD cases those individuals whose questionnaire-based symptoms related to shifts and did not occur in relation to holidays. The results of the field data showed that morning and night shifts induced SWD-related symptoms, and that symptoms during morning shifts in particular seemed to differentiate shift workers with and without SWD. In addition, our results point to poorer recovery from shift work in SWD. This was indicated by greater sleepiness during free time and less compensatory and light sleep on days off.

### Insufficient sleep

Earlier research on shift work has shown that shift work per se reduces TST related to night and early morning shifts (Sallinen and Kecklund 2010). Short sleep was also common in this study. The objective daily TST was ≤ 6 h among all the participants related to night shifts, and among those with SWD, also related to morning shifts.

Although objective measures of sleep length showed no significant differences in relation to any work shift or days off, the subjective daily TST was shorter before morning shifts among participants with SWD than among those without SWD. Previous studies using subjective sleep length measures averaged over different shift types have either associated (Kalmbach et al. 2015) or not associated (Di Milia et al. 2013) a decrease in TST with SWD. Instead of studying TST on a general level, we investigated it in relation to different shift types in real life. Our results add to the literature by indicating that subjectively evaluated TST seems to decrease before morning shifts in association with SWD.

Longer sleep latency, lower objective sleep efficiency, and poorer subjective sleep quality suggest that the quality of sleep remains lower among shift workers with SWD than among those without SWD. This is consistent with studies on shift systems that are distinct from the current study, showing poorer subjective sleep quality among swing-shift workers with SWD during a non-work period (Waage et al. 2009), and poorer subjective sleep efficiency among permanent night workers with SWD during night work periods (Gumenyuk et al. 2014) than among controls. In the current study, those with SWD showed rather good sleep quality, with less than 30 min of sleep latency (Sateia et al. 2017), over 85% sleep efficiency (Astill et al. 2013), and a rather good evaluation of quality of sleep. Despite this, both these objective and subjective measures indicated that shift workers with SWD had a poorer capacity to sleep than those without SWD, with respect to all studied shift types and days off.

Subjective sleep need was significantly greater among shift workers with SWD than among those without SWD. It is thus possible that longer sleep need predisposes to SWD. However, in cases of SWD, increased sleepiness and decreased sleep quality may also result in evaluations of longer individual sleep need. In addition, the sleep debt before morning shifts was greater among those with SWD, while compensatory sleep on days off was longer among those without SWD. This greater sleep debt may have resulted from poorer sleep quality, which can lead to slower recovery from work among individuals with SWD. Similarly, shorter sleep debt before morning shifts and the ability to sleep compensatory sleep on days off could have resulted from better sleep quality and subsequently sufficient recovery during the work period, in addition to days off, among the individuals without SWD.

We observed less light sleep on days off among participants with SWD than among those without SWD, which may reflect the difference between the groups’ compensatory sleep. For example, Jay et al. (2007) have shown that individuals sleep longer after sleep restriction, and that restriction of nightly recovery sleep reduces light and REM sleep. Although recovery sleep was not restricted in the current study, those with SWD may still have not had sufficient compensatory sleep, and would thus need longer to recover than those without SWD. The amount of SWS does not seem to associate with SWD, and based on our results, the poorer recovery of the participants with SWD is more likely to be related to less efficient sleep.

Further, in the current study, shift workers with SWD scored higher points on the bedtime stress scale than those without SWD in association with all shift types and days off. Interestingly, higher job strain in shift work has shown to associate with difficulties in initiating sleep (Karhula et al. 2013), a symptom characteristic to SWD. Sleep-reactivity (i.e. sensitivity of sleep to stress) has been shown to predict SWD (Kalmbach et al. 2015). If the sleep-reactivity in the current study was higher among those with SWD than among those without SWD, the slightly but significantly higher points in the bedtime stress scale could have contributed to the manifestation of the symptoms of SWD, although those with SWD were rather calm and relaxed.

### Alertness and sleepiness

We observed more performance lapses in PVT among participants with SWD than among those without SWD at the beginning of night shifts, whereas subjective sleepiness was greater at the end of night shifts, reaching a high level (KSS > 6) (Åkerstedt et al. 2014) among the participants with SWD. The greatest daily sleepiness was high in relation to night and morning shifts. In fact, subjective sleepiness, either the last 5 min KSS or the greatest daily KSS, was greater among the participants with SWD in relation to all the studied shifts, and showed a similar tendency on days off. Regarding night shifts, our findings are consistent with a laboratory study that showed increased night-time sleepiness in association with SWD among permanent night workers (Gumenyuk et al. 2014). However, findings from laboratory studies on permanent night work cannot be extrapolated to real-life shift work as such. To conclude, the current study supports increased sleepiness in association with SWD among shift workers in several shift types.

### Differences in flexibility, chronotype, physical exercise, and caffeine consumption

In this study, shift workers with SWD had poorer flexibility in their sleeping habits, which is supported by one epidemiological study (Flo et al. 2012), but unsupported by another (Di Milia et al. 2013). Furthermore, the prevalence of evening chronotype was higher among participants with SWD. Earlier research has both associated (Asaoka et al. 2013) and not associated (Taniyama et al. 2015) evening chronotype with SWD.

As a stimulant, caffeine can disrupt sleep (Wright et al. 2013). Participants with SWD consumed less caffeinated drinks and engaged in physical exercise more often than those without SWD. Individuals reacting with poor sleep to situational stressors such as shift work may also be sensitive to the sleep-disturbing effects of caffeine (Bonnet and Arand 2003) and may avoid consuming it before bedtime. This may have diminished the consumption of caffeine in the current SWD group. Further, exercise can adjust and improve circadian rhythm and sleep (Schroeder and Colwell 2013). However, if poorly scheduled, exercise can disturb sleep, which may have contributed to the impaired sleep and alertness among those with SWD.

### Practical relevance—assessment of SWD

Shift work misaligns circadian and diurnal rhythms, almost inevitably causing sleep disturbances. This is a challenge for most shift workers, not just for those with SWD. Due to inefficiency or rigidity in sleeping, shown by less compensatory sleep and less light sleep on days off, generally poorer sleep quality (according to sleep diaries and actigraphy), and less flexibility in sleeping habits, individuals with SWD may not be able to optimally utilize the recovery periods allowed by their rosters. This can degrade alertness and increase the risk of SWD. In fact, the quality of sleep among those without SWD appeared rather high, and this may be essential for coping with recurring disturbance to circadian rhythms.

Our results support the practice of screening the symptoms in naturalistic settings in association with both work shifts and days off since symptoms can appear at different points of time. In this study, the subjective SWD symptoms were more abundant than the objective findings. However, although the current study offers no suggestion of, for example, threshold values, the subjective quality of sleep and sleepiness appear to be useful measures as a first indication of SWD.

### Limitations

We did not diagnose SWD by a clinical evaluation, but used a questionnaire to define the disorder, and field measurements to verify it. We were not able to consider all the established factors affecting insomnia and/or sleepiness in this study. However, health conditions with insomnia symptoms and/or sleepiness also typically induce these symptoms while on holiday. The exclusion of volunteers with significant holiday-related symptoms of insomnia and/or sleepiness was likely to reduce the bias due to the diagnosed and non-diagnosed conditions influencing these symptoms.

We verified a reduction in TST in comparison to that on days off in the field data. And although the ICSD-3 does not require verification of reduced TST after a shift work washout period, examining TST after a longer recovery period could have verified whether the SWD cases’ TST had really decreased. However, subjective shift-related TST decreased by approximately 1–2 h in the SWD group when compared to sleep need, bearing in mind that ‘sleep need’ is not equal to ‘TST after a washout period’.

On days off, the participants were given time between 10:00 and 13:00 to complete the PVT, because vigilance performance has shown to be rather stable during this time (Monk et al. 1997). Since cognitive performance follows circadian rhythm it is possible that it may have changed during these three hours (Mollicone et al. 2010).

Because our aim was to provide a detailed picture of sleep and alertness among shift workers who had a SWD compared to those who had none, we included only clear cases and clear non-cases of SWD based on our definition. This increased the contrast between the groups. In our opinion, the inclusion of those with ‘milder SWD symptoms’ in the SWD group would have blurred the comparison since it is unclear whether or not they actually had SWD.

Those with SWD were younger than those without SWD. Young age has been related to shift work tolerance (Saksvik et al. 2011). This may have diminished some group differences, as older age is known to unfavorably affect sleep and recovery (Costa and Sartori 2007; Kiss et al. 2008). Thus, smaller group differences would likely have become significant in a large sample. Nevertheless, statistical power was limited due to small sample size. Moreover, although age and alternatively chronotype were included in the LMMs, we were not able to adjust other analyses for age or chronotype. Similarly, analyses were not adjusted for other possible confounders. In future studies, larger sample sizes could verify our findings. In addition, caution should be used when generalizing our results to shift systems that were not included in this study, for example permanent night work.

### Strengths

We based the definition of SWD on the shift-type specific occurrence of insomnia symptoms and sleepiness. As SWD symptoms should associate with work schedules overlapping the usual time for sleep, and recovery from a schedule can take several days, to qualify as having SWD in our study, the participant had to report decreased SWD symptoms after 2 weeks on holiday. All the participants that our screening method classed as positive for SWD also showed a disturbed sleep and wake pattern via both sleep diaries and actigraphy, as required by the ICSD-3. This is also the first study to comply with the ICSD-3’s requirement of reduced TST, verified after defining SWD. Many of the screening methods previously used to define SWD have not been as suitable as our questionnaire. For example, not all studies on SWD have associated SWD symptoms with work schedules that temporally overlap habitual sleep (Di Milia et al. 2013). Our study is the first to use EEG-based measures of sleep or PVT in a field setting for studying SWD. In addition, our study groups were also comparable in terms of shift characteristics, which reduced potential bias deriving from these factors.

## Conclusions

The current study adds to the knowledge on the manifestation of SWD in real-life morning, evening, and night shifts and days off in several ways. Those with SWD reported sleeping less and had greater sleep debt than those without SWD before morning shifts, which is something that the current study is the first to show. Moreover, this is the first time that less compensatory sleep has been shown among those with SWD than among those without SWD during days off. We were also able to show that shift workers with SWD were sleepier than those without SWD, also in relation to morning and evening shifts. In addition, shift workers with SWD had both objectively and subjectively poorer quality of sleep and a consistently lower level of relaxation at bedtime than those without SWD. The combination of above changes in sleep and alertness with less compensatory sleep on days off and poorer flexibility in sleeping habits may have detrimental effects for a shift worker.

## Electronic supplementary material

Below is the link to the electronic supplementary material.

Supplementary material 1 (PDF 10 KB)
