# Supplementary material for: Sleep and alertness in shift work disorder: findings of a field study
Source: Int Arch Occup Environ Health. 2018 Dec 3;92(4):523–33. doi: 10.1007/s00420-018-1386-4 (PMC6435614; doi:10.1007/s00420-018-1386-4)
Supplement: Supplementary file 1 — Supplementary material 1 (PDF 10 KB) [file 420_2018_1386_MOESM1_ESM.pdf]

Sleep and alertness in shift work disorder – findings of a field study

International Archives of Occupational and Environmental Health

Päivi Vanttola[1,2], Mikko Härmä[1], Katriina Viitasalo[3], Christer Hublin[1], Jussi Virkkala[1], Mikael Sallinen[1,4], Kati Karhula[1], Sampsa Puttonen[1]

[1] Finnish Institute of Occupational Health, Finland; [2] Faculty of Medicine, University of Helsinki, Finland; [3] Finnair Health Services, Finland; [4] Department of Psychology, University of Jyväskylä, Finland

E-mail: paivi.vanttola@ttl.fi

Supplementary material

Supplementary table 1 Shift-specific questions on insomnia and sleepiness

Questions/symptoms	Sub-questions	never/ rarely	rather rarely	rather often	often/ continuously	not applicable
1. How often do you experience difficulties in initiating sleep in relation to	i) morning shifts?	( )	( )	( )	( )	( )
	ii) evening shifts?	( )	( )	( )	( )	( )
	iii) night shifts?	( )	( )	( )	( )	( )
	iv) two weeks of being on holiday?	( )	( )	( )	( )	( )
2. How often do you awaken during a sleep period in relation to	i) morning shifts?	( )	( )	( )	( )	( )
	ii) evening shifts?	( )	( )	( )	( )	( )
	iii) night shifts?	( )	( )	( )	( )	( )
	iv) two weeks of being on holiday?	( )	( )	( )	( )	( )
3. How often do you experience difficulties falling asleep after awakening in relation to	i) morning shifts?	( )	( )	( )	( )	( )
	ii) evening shifts?	( )	( )	( )	( )	( )
	iii) night shifts?	( )	( )	( )	( )	( )
	iv) two weeks of being on holiday?	( )	( )	( )	( )	( )
4. How often do you experience non-restorative sleep in relation to	i) morning shifts?	( )	( )	( )	( )	( )
	ii) evening shifts?	( )	( )	( )	( )	( )
	iii) night shifts?	( )	( )	( )	( )	( )
	iv) two weeks of being on holiday?	( )	( )	( )	( )	( )
5. How often do you experience daytime sleepiness in relation to	i) morning shifts?	( )	( )	( )	( )	( )
	ii) evening shifts?	( )	( )	( )	( )	( )
	iii) night shifts?	( )	( )	( )	( )	( )
	iv) two weeks of being on holiday?	( )	( )	( )	( )	( )
6. How often do you experience difficulties in staying awake at work in relation to	i) morning shifts?	( )	( )	( )	( )	( )
	ii) evening shifts?	( )	( )	( )	( )	( )
	iii) night shifts?	( )	( )	( )	( )	( )

Criteria for SWD and non-SWD groups: SWD cases had to report at least four of the symptoms (Questions 1–5) 'never/rarely' or 'rather rarely' after two weeks on holiday (iv). In addition, they had to have at least one of the 'never/rarely' or 'rather rarely' occurring symptoms (Questions 1–5) or difficulties in staying awake at work (Question 6) 'often/continuously' in connection with some shift (i – iii). The non-SWD group was composed of those who reported at least five of the six symptoms as 'never/rarely' or 'rather rarely' in relation to all shifts (Questions 1–6) and on holiday (Questions 1–5). Those who reported at least four of the five symptoms (Questions 1-5) as 'never/rarely' or 'rather rarely' in relation to all shifts (Questions 1–5) and on holiday (Questions 1–5) were also included in the non-SWD group. In this case, they had to 'never/rarely' or 'rather rarely' have difficulties in staying awake at work in relation to all shifts (Question 6: i – iii).

Supplementary table 2 Work shift characteristics of the participants having morning shifts (n = 30), evening shifts (n = 31), and night shifts (n = 26)

	Median	(IQR)
Morning shifts		
Monthly number	6.3	(3.3)
Shift start time (h:mm)	6:00	(0:19)
Shift end time (h:mm)	15:46	(1:14)
Evening shifts		
Monthly number	4.1	(1.7)
Shift start time (h:mm)	14:00	(0:30)
Shift end time (h:mm)	00:00	(0:40)
Night shifts		
Monthly number	5.1	(1.5)
Shift start time (h:mm)	21:15	(2:30)
Shift end time (h:mm)	6:00	(0:30)
Number of consecutive		
Days off	2.0	(0.5)
Morning shifts	2.0	(1.5)
Evening shifts	2.0	(2.0)
Night shifts	2.0	(2.0)

Supplementary table 3 Fragmentation index of sleep among shift workers with and without SWD

	SWD group			Non-SWD group			Group[a]		
	Mean	(SD)	n	Mean	(SD)	n	df	F	p
Days off	25	(8)	22	17	(8)	9	27.970	4.00	0.055
Before morning shifts	28	(13)	22	19	(5)	8	28.585	3.94	0.057
After evening shifts	25	(10)	22	21	(7)	9	23.210	0.91	0.349
After night shifts	21	(9)	18	18	(9)	8	21.481	1.00	0.328

[a] Linear mixed model for repeated measurements with Group and Age as main effects
